# Influence of Birth Preparedness, Decision-Making on Location of Birth and Assistance by Skilled Birth Attendants among Women in South-Western Uganda

**DOI:** 10.1371/journal.pone.0035747

**Published:** 2012-04-27

**Authors:** Jerome K. Kabakyenga, Per-Olof Östergren, Eleanor Turyakira, Karen Odberg Pettersson

**Affiliations:** 1 Department of Clinical Sciences, Social Medicine and Global Health, Lund University, Malmö, Sweden; 2 Department of Community Health, Mbarara University of Science and Technology, Mbarara, Uganda; Universidad Peruana Cayetano Heredia, Peru

## Abstract

**Introduction:**

Assistance by skilled birth attendants (SBAs) during childbirth is one of the strategies aimed at reducing maternal morbidity and mortality in low-income countries. However, the relationship between birth preparedness and decision-making on location of birth and assistance by skilled birth attendants in this context is not well studied. The aim of this study was to assess the influence of birth preparedness practices and decision-making and assistance by SBAs among women in south-western Uganda.

**Methods:**

Community survey methods were used to identify 759 recently delivered women from 120 villages in rural Mbarara district. Interviewer-administered questionnaires were used to collect data. Logistic regression analyses were conducted to assess the relationship between birth preparedness, decision-making on location of birth and assistance by SBAs.

**Results:**

35% of the women had been prepared for childbirth and the prevalence of assistance by SBAs in the sample was 68%. The final decision regarding location of birth was made by the woman herself (36%), the woman with spouse (56%) and the woman with relative/friend (8%). The relationships between birth preparedness and women decision-making on location of birth in consultation with spouse/friends/relatives and choosing assistance by SBAs showed statistical significance which persisted after adjusting for possible confounders (OR 1.5, 95% CI: 1.0–2.4) and (OR 4.4, 95% CI: 3.0–6.7) respectively. Education, household assets and birth preparedness showed clear synergistic effect on the relationship between decision-maker on location of birth and assistance by SBAs. Other factors which showed statistical significant relationships with assistance by SBAs were ANC attendance, parity and residence.

**Conclusion:**

Women’s decision-making on location of birth in consultation with spouse/friends/relatives and birth preparedness showed significant effect on choosing assistance by SBAs at birth. Education and household assets ownership showed a synergistic effect on the relationship between the decision-maker and assistance by SBAs.

## Introduction

Assistance by skilled birth attendants (SBAs) is one of the strategies aimed at reducing maternal morbidity and mortality in low-income countries such as Uganda [1], [2], [3], [4]. Most of the causes of maternal morbidity and mortality are preventable and attributed to the three delays; delay to make a decision to seek care, delay to reach the place of care, and delay to receive appropriate care [5]. Having a skilled birth attendant (SBA) at every delivery has been found to markedly reduce maternal morbidity and mortality in countries such as Malaysia and Sri Lanka [4], [6].

Prompt decision-making is a prerequisite for reducing delay to seek care [7], [8]. Decision-making on utilization of health services is related to women’s autonomy, which is defined by Hindin as women’s ability to make decisions in the household [9], [10]. Studies have indicated that in some sub-Saharan countries men generally are decision makers regarding the location at which their spouse should give birth [11], [12], [13], [14], [15], [16], whereas in south East Asia it is mostly mother-in-laws who determine the location of birth [17]. At the International Conference on Population and Development held in Cairo, Egypt in 1994, it was affirmed that when women are empowered to make own decisions they would access health services more quickly [18]. However, some studies have indicated that the association between women’s decision-making and utilisation of maternal health services is not convincing [19], [20]. Conversely joint decision-making between women and their spouses have shown increased uptake of maternal health services in Nepal [10] and Uganda [21].

Birth preparedness, which comprises identification of skilled birth attendant, health facility, saving money, identifying transport and making other preparations for childbirth is aimed at increasing assistance by SBAs and also reducing delay in reaching place of care. Studies have indicated that women who are birth prepared are more likely to be assisted by skilled birth attendants [22], [23], [24]. Observations have been made that those countries, which have managed to reduce maternal mortality drastically in the last 50 years, such as Malaysia and Sri Lanka, have invested much in increasing the number of SBAs and access to emergency obstetric care [25].

In year 2000 the millennium summit introduced the eight Millennium Development Goals (MDGs) to achieve development in low-income countries [26]. Two of the eight goals (4 and 5), which directly relate to child and maternal health, are commonly referred to as health MDGs. Of the 68 countries that were being monitored regarding coverage of interventions, only 16 are on track to achieve the MDG targets by the year 2015. Moreover most of the countries reported to be on track to achieve MDG targets are located in North Africa, South America and Asia [27]. In the same study Uganda’s progress towards achieving the health MDGs was categorised as insufficient. Likewise the latest Uganda MDG report (2010) indicated that the progress to achieve the health MDGs was slow and that more effort is needed in order for the country to attain the set targets by 2015 [28].

Since 2008 the Uganda government has been implementing a road map aimed at accelerating the reduction of maternal and newborn mortality and morbidity [29]. One of the strategies outlined in the road map is to empower communities to ensure a continuum of care between the household and the health care facility. Two of the factors, which affect this continuum of care, are decision-making and birth preparedness at the household level. Few studies nationally as well as internationally have reported on the relationship between birth preparedness practices, decision-making on location of birth and assistance by SBAs. The aim of this study was to assess the influence of birth preparedness practices and decision-making on location of birth and assistance by SBAs among women in south-western Uganda.

## Methods

### Study Design and Setting

A community survey of recently delivered women (within the last 12 months) and currently pregnant women was conducted between September 2010 and May 2011 in Mbarara district. Mbarara district is located in the south-western region of Uganda. The district covers an area of 1788 square kilometres, and had a projected population for 2010 of 427,200 with 80% working and residing in rural areas. Administratively the district is divided into three counties (health sub-districts); Mbarara municipality, which is the main urban centre while Kashari and Rwampara counties are largely rural with few small towns/trading centres [30]. Most of the land terrain in Kashari is flat with a few areas covered by hills and has better road infrastructure than Rwampara, which has a hilly terrain. The majority of the population in the district are Banyankole by tribe and mostly depend on subsistence agriculture for their livelihood.

Mbarara regional referral hospital, located in Mbarara town, is the only public hospital, which provides emergency comprehensive obstetric care services in the district. The hospital also serves as the main referral and teaching health facility for the south-western region and Mbarara University respectively. There are three private hospitals, all located in Mbarara municipality, one is purely private while the other two are owned by religious organisations and are categorised as private not for profit (PNFP). The district has 47 health centres of levels II-IV, which among other basic outpatient and inpatient health services offer maternal health care services (antenatal, natal and postnatal). Kashari County has eight health centres level III and IV, which offer delivery services while Rwampara has only four. The most recent Uganda Demographic and health survey of 2006 indicates that only 32% of women residing in rural areas deliver under the care of skilled birth attendants compared to 80% of their urban counterparts [31].

### Sampling Method and Sample Size

Two-stage cluster sampling was used to select study participants for a broader study on pregnancy and childbirth. In the first stage, a list of villages and the respective number of households was used to independently select 58 villages in Kashari and 62 Rwampara counties. In total, one hundred and twenty villages were randomly chosen. In the second stage, households in which there was a woman who had recently delivered or currently was pregnant were consecutively identified with the assistance of village health team members (community health workers). In each village, the first ten women who met the study criteria were interviewed. In case there were two or more qualifying women in one household, only one was chosen at random for the interview. The number of women to be interviewed per village was fixed to 10, which was regarded by the study team as the optimum number two research assistants could interview per day. Compared to increasing the number of respondents per cluster, increasing the number of clusters/villages is also a more efficient approach to increasing study power in a cluster survey. In total 1199 recently delivered and currently pregnant were interviewed during the survey. Only one woman declined to participate in the survey. The focus of this particular study being birth preparedness and decision-making on location of birth and assistance by SBAs, and therefore this paper only included information from the 759 women who had delivered within 12 months prior the date of the survey and had complete data on the outcome of interest (assistance by skilled birth attendant). Data from 5 women were excluded from analysis as it lacked information on the outcome of interest. Data from currently pregnant women were consequently not included in this study.

### Data Collection and Management

A safe motherhood questionnaire developed by the Maternal Neonatal Program of JHPIEGO, an affiliate of John Hopkins University was used [32]. It contained four sections namely; socio-demographic information and reproductive history, knowledge on pregnancy and childbirth, experiences related to last pregnancy and childbirth, and exposure to media and interventions. The questionnaire was adapted to fit the Ugandan context and subsequently pretested in the neighbouring district of Isingiro. Minor modifications were made after pretesting. Twelve research assistants (all social sciences graduates) with experience in survey data collection were trained for one week, participated in the pretesting and thereafter conducted the interviews under supervision. The fieldwork to collect data was completed in 25 days. During data collection all questionnaires were checked for completeness and consistency by the field supervisors. All data were coded, and double entered into a database and validated using Epi Data Version 3.1. Data clerks verified all data entry mismatches and made corrections in the database. Further cleaning was done using Stata Version 9 (Stata Corp, Texas).

### Definition of Variables

#### Socio-demographic variables

County of residence was coded “Kashari” or “Rwampara”. Location of residence was categorised as “rural” or “semi-urban”. Age was categorised into 2 groups “<25″ (young women 16–24), “≥25″ (older). Marriage was dichotomised so that “married/in union” was coded “married” and “single”, “widowed”, “divorced”, “separated” was coded “not married”. Highest education level completed was dichotomized so that “no formal schooling”, “primary” were coded into “<Secondary” and any education beyond primary was coded “≥Secondary”. Occupation was dichotomized so that “commercial farmer”, “trader”, “salaried employment”, were coded “regular income” while “house wife”, “casual labourer” were coded “irregular income”. Household assets ownership (radio, television set, mobile phone, bicycle) were scored (1,2,3,4) so that ownership of two or more assets was classified “high” and ownership of one or none coded “low”. Travel from health facility with delivery services was coded “<1 hour” (near) and “≥1 hour” (far).

#### Reproductive and decision making variables

Antenatal Care (ANC) attendance - was coded “less than 4 times” (<4) and “four or more times” (≥4). The variable “Parity” was divided into three groups of “1″, “2–4″, “≥5″. Birth preparedness: A woman was classified as “well birth prepared” in the most recent pregnancy if she had accomplished three of the following practices: identified skilled health professional, saved money, identified transport or had delivery kit/materials. A woman who made arrangements for birth in less than three of the four practices was classified as “not well birth prepared”.

Decision-maker on location of birth: A recently delivered woman was asked the question “Who made the final decision about where you would give birth?” The respondents’ spontaneous responses were categorized into: “respondent alone”, “husband”, “respondent and husband”, “others”.

#### Outcome variable

Assistance by a skilled birth attendant (SBA) in the most recent birth was the outcome variable. WHO defines a skilled birth attendant as “an accredited health professional – such as a midwife, doctor or nurse – who has been educated and trained to proficiency in the skills needed to manage normal (uncomplicated) pregnancies, childbirth and the immediate postnatal period, and in the identification, management and referral of complications in women and newborns” [33]. Women were asked “Who assisted with their most recent birth?” and spontaneous responses were recorded. Responses of “doctor, midwife/nurse”, “clinical officer” (equivalent of assistant physician) were classified as assisted by SBAs and coded 1. Assistance by traditional birth attendant, relative or friend, or any other person who is not a health professional based on the respondent’s description was classified as unskilled birth attendant and assigned code of 0.

### Statistical Analysis

All analysis accounted for the intra-cluster correlation. For selected characteristics, the proportion of participants was computed using Stata’s SVY commands. The odds of assistance by skilled birth attendant at birth were compared between categories of the independent variables using multilevel random intercept logistic regression model, the village being the grouping variable. Odds ratios and 95% confidence intervals were obtained. Multicollinearity was assessed using the stata’s *Collin* command. The variance inflation factor for the predictors of interest was less than 2, indicating no multicollinearity. Variables whose association to assistance by SBAs was statistically significant or when an association was expected and p-value of less than 0.2, were included in multivariable analysis, a rather generous measure to assure that even rather weak confounding effects could be accounted for. Stepwise multivariable random effects logistic regression with a random intercept was carried out to determine motivating factors for assistance by SBAs and adjusted for known confounders. The potential effect modification of age, education, household assets ownership, birth preparedness on the association between decision-making and assistance by skilled birth attendants was assessed using “the departure from additivity model” [34]. Statistical analysis was performed using Stata Version 9.

### Ethical Considerations

The Uganda National Council of Science and Technology granted ethical clearance for the study the study. Permission was also sought from local leaders at the district, county and village levels. Participants in the study gave individual written informed consent.

## Results

Seven hundred and fifty nine women who had delivered within twelve months prior to the start of the survey were included in the study. The sample had an age range of 16 to 45 years (mean 27+/- 6 years). Table 1 shows the socio demographic, reproductive, birth preparedness and decision-making on location of birth characteristics of the sample. Most respondents were living in rural areas (77%), were married (95%), had lower than secondary education (75%) and had irregular income (76%). The majority (73%) were from households, which owned at least two household assets (radio, television set, mobile phone, bicycle) and resided less than one-hour travel time to health facilities offering delivery services. Sixty eight per cent had parity of one to four and 68% had four or more ANC attendance visits. Thirty five per cent of the women were birth prepared while 65% were not. The final decision on location of birth was taken by the woman herself (36%), woman in consultation with spouse (56%) and woman together with other relative or friend (8%).

**Table 1:** Socio-demographic, reproductive characteristics, birth preparedness and decision-maker on location of birth characteristics (N = 759).

Characteristic	n	(%)
**County**		
Kashari	387	51.8
Rwampara	372	48.2
**Location of residence**		
Rural	636	77.1
Semi-urban (small town)	123	22.9
**Age (years)**		
<25	301	39.6
≥25	458	60.4
**Marital status**		
Not married	37	4.6
Married	721	95.4
**Education level**		
Less than secondary (low)	583	75.2
Secondary and above (high)	175	24.8
**Occupation**		
Irregular income	600	76.4
Regular income	157	23.6
**Household assets ownership**		
Low (0–1)	212	26.9
High (2+)	547	73.1
**Travel time to health facility**		
< 1 hour	416	60.1
≥1 hour	336	39.9
**Parity**		
1–2	307	40.3
≥3	452	59.7
**ANC Attendance**		
<4 visits	245	32.3
≥ 4 visits	514	67.7
**Gestation age at first ANC clinic**		
1–3months	302	39.4
≥4 months	446	60.6
**Birth preparedness**		
Not well prepared	488	64.7
Well prepared	271	35.3
**Decision-maker on location of birth**		
Woman alone	271	35.8
Spouse +/- respondent	426	55.9
Family/friend	62	8.2

Of the 759 recently delivered women, 68.5% were assisted by SBAs while 29.1% were assisted by non-SBAs and 2.4% had solitary births. Furthermore 67% of the deliveries were conducted in health facilities while 31% took place at home or traditional birth attendant’s residence.

Table 2 shows the association between socio-demographic characteristics, reproductive characteristics, birth preparedness, decision-maker on location of birth and assistance by SBAs at birth. Women, who were residing in Rwampara County, were less likely to be assisted by SBAs than those residing in Kashari County (OR 0.6, 95% CI 0.4–0.9). The location of residence, whether rural or semi-urban, appeared to influence the choice of assistance by SBAs. Women whose residences were located in small towns were more likely to be assisted by SBAs compared to those whose residences were rural (OR 2.6, 95% CI: 1.4–4.8) The level of education appeared to influence use of skilled birth attendant services and in the study sample, women who had secondary education or above were more likely to choose assistance by SBAs than those who had lower levels of education (OR 3.0, 95% CI: 1.9–4.8). Women whose household assets ownership scored high were more likely to choose assistance of SBAs than those with low household assets score (OR 2.1, 95% CI: 1.5–3.1). The time taken to reach a health facility also appeared to influence the use of SBAs. Women who were residing at a distance of more than one-hour travel time from a health facility offering childbirth services were less likely to choose assistance by skilled birth attendant (OR 0.7, 95% CI: 0.5–1.0).

**Table 2:** Association (OR 95% CI) between socio-demographic variables, reproductive variables, birth preparedness, decision-maker on location of birth and assistance by skilled birth attendants (N = 759).

Characteristic	Assisted by skilled birth attendantn (%)	OR (95% CI)
**Health sub-district**		
Kashari	275 (74.2)	1.0
Rwampara	225 (61.9)	0.6 (0.4–0.9)
**Type of residence**		
Rural	401 (63.4)	1.0
Semi-urban (small town)	99 (85.1)	2.6 (1.4–4.8)
**Age (years)**		
<25	206 (70.5)	1.0
≥25	294 (66.9)	0.7 (0.5–1.0)
**Marital status**		
Married	479 (68.6)	1.0
Not married	21 (63.0)	0.6 (0.3–1.3)
**Education level**		
Less than secondary	357 (63.0)	1.0
Secondary+	143 (84.5)	3.0 (1.8–4.8)
**Occupation**		
Not a regular income	387 (65.3)	1.0
Regular income	113 (78.2)	1.4 (0.9–2.2)
**Household asset ownership score**		
Low	112 (55.5)	1.0
High	388 (73.1)	2.1 (1.5–3.1)
**Time to nearest health facility**		
<1 hour	297 (73.8)	1.0
≥1 hour	203 (60.1)	0.7 (0.5–1.0)
**Parity**		
1–2	226 (76.5)	2.2 (1.5–3.1)
≥3	274 (62.9)	1.0
**ANC attendance**		
Less than 4 times	132 (57.2)	1.0
4 or more times	368 (73.6)	2.2 (1.5–3.1)
**Gestation age at first ANC clinic**		
1–3months	283 (72.6)	1.4 (1.0–1.9)
≥4 months	212 (66.2)	1.0
**Birth preparedness**		
Not well prepared	299 (63.7)	1.0
Well prepared	197 (76.8)	2.1 (1.4–3.1)
**Decision-maker on location of birth**		
Woman alone	126 (51.8)	1.0
Spouse +/- respondent	329 (78.0)	5.5 (3.7–8.4)
Other relative	45 (74.3)	4.6 (2.3–9.5)

The parity of the woman appeared to influence the choice of assistance by SBAs. Women who had parity of one or two were more likely to choose assistance by SBAs than those who had parity of five or more (OR 2.2, 95% CI: 1.5–3.1). Women who had their first antenatal clinic visit with a gestation age of 1–3 months were more likely to choose assistance of skilled birth attendant than those who had their first ANC visit at gestation age of 4 months or above (OR 1.4, 95% CI: 1.0–1.9). Those women who attended ANC 4 times or more were more likely to choose assistance by skilled birth attendant at the time of delivery than those who had less than four ANC visits (OR 2.2, 95% CI: 1.5–3.1).

Birth preparedness was also associated with being assisted by skilled birth attendant at the time of delivery. Women who were well prepared for childbirth were more likely to choose assistance by SBAs than those who were not well prepared (OR 2.1, 95% CI: 1.4–3.1). In this study the person who made the final decision on the location of birth was significantly associated with assistance by SBAs. In cases where the women made the final decision in consultation with their husbands the likelihood of choosing assistance by SBAs was significantly higher than cases in which women made the decision on location of birth alone (OR 5.5, 95% CI: 3.7–8.4). Similarly when other people, mostly relatives, were consulted in making the final decision on location of birth, the likelihood of choosing assistance by SBAs was greater than when women made the decision on their own (OR 4.6, 95% CI: 2.3–9.5).

Table 3 shows the result of multivariable logistic regression analyses performed to account for possible confounding regarding the association between independent variables of interest; “birth preparedness”, “the decision-maker on location of birth” and the outcome variable “assistance by SBAs”. The variables ANC attendance, parity, education level attained, household assets ownership, residence (rural or semi-urban) and county were introduced in three groups in a step-wise manner. In model one ANC attendance and parity were adjusted for while in model two, highest education level attained and household assets ownership were introduced. Subsequently in model three the location of residence (rural or semi-urban) and the county were added to the model. In model one the introduction of ANC attendance and parity showed marginal effect on the association between decision-maker on location of birth/birth preparedness and assistance by SBAs. However in Model 2 and 3 there was a minimal confounding noticed between the main variables of interest and potential confounding variables were introduced in the two steps.

**Table 3:** Association (Odds Ratios, 95% Confidence Intervals) between birth preparedness, decision-maker on location of birth and assistance by skilled birth attendants: Results of multivariable logistic regression analyses.

Factors	Model 1Adjusted for ANC, Parity	Model 2Adjusted for ANC, Parity, Education, Assets	Model 3Adjusted for ANC, Parity, Education, Assets, Residence, County
Birth preparedness: *Well prepared for birth vs not well prepared*	1.6 (1.1–2.5)	1.5 (1.0–2.3)	1.5 (1.0–2.4)
Decision-maker: *With others vs Respondent herself*	4.7 (3.1–7.2)	4.3 (2.9–6.6)	4.4 (3.0–6.7)

Further analyses were performed to check for possible effect modification of age, education, household assets ownership, birth preparedness on the relationship between decision-making and choosing assistance by skilled birth attendant **[** **Table 4** **]**. The analyses revealed that high level of education, being birth prepared and having high household assets ownership all showed synergistic effect regarding the association between decision-maker on location of birth and assistance by SBAs.

**Table 4:** Analysis of effect modification between age, education, household assets ownership, birth preparedness and decision-maker on location of birth regarding assistance by skilled birth attendants.

	Assistance by skilled birth attendants (N = 500)	
	n (%)	OR (95% CI)
**Age and decision-maker on location of birth**		
<25 years/respondent made decision	50 (10.0)	1.0 (ref)
<25 years/spouse & respondent or someone else	156 (31.2)	5.0 (2.7–9.4)
≥25 years/respondent made decision	76 (15.2)	0.7 (0.4–1.2)
≥25 years/spouse & respondent or someone else	218 (43.6)	3.7 (2.1–6.7)
**Education and decision-maker on location of birth**		
Less than Secondary education/respondent made decision	91 (18.2)	1.0 (ref)
Less than secondary education/spouse & respondent or someone else	266 (53.2)	5.4 (3.5–8.4)
≥Secondary education/respondent made decision	35 (7.0)	3.2 (1.5–6.9)
**Household assets ownership and decision-maker on location of birth**		
Low household asset score/respondent made decision	39 (7.8)	1.0 (ref)
Low household asset score/spouse & respondent or someone else	73 (14.6)	4.4 (2.2–8.6)
High household asset score/respondent made decision	87 (17.4)	1.6 (0.9–2.8)
High household asset score/spouse & respondent or someone else	301 (60.2)	8.2 (4.7–14.4)
**Birth preparedness and decision-maker on location of birth**		
Not well prepared/respondent made decision	99 (19.8)	1.0 (ref)
Not well prepared/spouse & respondent or someone else	203 (40.6)	4.2 (2.6–6.7)
Well prepared/respondent made decision	27 (5.4)	1.1 (0.6–2.2)
Well prepared/spouse & respondent or someone else	171 (34.5)	8.7 (5.0–15.7)

## Discussion

Our results showed a clear relationship between decision-maker on location of birth and choosing assistance by skilled birth attendant among women in south-western Uganda. Similarly there was a significant association between birth preparedness and assistance by SBAs. The relationships remained statistically significant even after controlling for the potential confounding of ANC attendance, parity, education level attained, household assets ownership, place of residence (rural or semi-urban) and county.

Findings from our study indicate that, when women made the final decision on location of birth in consultation with either the spouse or other people, the likelihood of giving birth assisted by a skilled birth attendant was very high. However, when women made the final decision alone; the likelihood of giving birth assisted by SBAs was significantly reduced. This finding may be related to the desire expressed by women to protect their own integrity as reported in a qualitative study conducted on the community’s perception of obstructed labour in Mbarara district [35]. In the said study the authors reported that women prefer solitary births in order to be in control of the childbirth process and to avoid being exposed to strangers. They only involved others (husbands, relatives, traditional birth attendants) when they encountered complications. However in our study women who made joint decisions with spouses or other close persons (mother in laws, friends) on the other hand were more likely to deliver under the assistance of skilled birth attendant. Joint decision-making was further strengthened by high education and high household assets ownership.

Internationally, particularly in lower income countries (LICs), there is a growing awareness of the need to involve men in all stages of pregnancy, childbirth and postnatal education programmes in order to achieve the MDG 5 thereby reducing maternal mortality [15], [36]. Our finding therefore not only calls for review of the way antenatal care is carried out where the current approach is mostly targeting the pregnant women but also to discuss with the community directly about interventions to increase male involvement.

Contrary to our findings, Amooti-Kaguna and Nuwaha, Kyomuhendo and Merchant reported from their studies in Uganda that men decide the places where their spouses should give birth from [11], [37], [38]. However, these studies did not explore the relationship between decision-making on location of birth and assistance by SBAs. A study conducted in Nepal on the other hand showed that spousal discussion of family planning was associated with increased likelihood of receiving both antenatal and delivery care [20]. Studies conducted elsewhere have shown minimal effect on the association between women’s decision-making and assistance by skilled birth attendant [19], [39].

In our study birth preparedness was significantly associated with assistance by SBAs in recently delivered women. Being birth prepared is most likely an indication that the women are consciously aware of the risks inherent in pregnancy and childbirth hence the significant use of skilled birth attendant services. Our results also support findings from earlier studies conducted in Uganda, which have reported a significant increase in use of health facilities during deliveries by women who are birth prepared [15], [22]. Similarly authors in other parts of the world have reported association between birth preparedness and being assisted by SBAs [24], [40].

In this study higher education and high household assets ownership were found to have synergistic effect on the relationship between women’s decision-making in consultation with spouse/friends/relatives and assistance by skilled birth attendant. Education promotes better understanding of health messages, which empowers women to improve not only their health but also of their families, as they become enabled to make informed choices [17], [41]. Similarly the level of household assets ownership, which was a proxy for level of income in our study, determines which women access and utilise maternal health services. Studies elsewhere have indicated significant association between high education, high income and increased levels of assistance by skilled birth attendants among women [38], [42], [43], [44].

Other factors, which were identified in our study to be significantly associated with choosing to be assisted by SBAs, include ANC attendance, parity, residence and county. Antenatal care visits provide opportunity for expectant mothers to be assessed for possible risks and also provides an opportunity for women to be educated on use of maternal health care services. Women who had at least four ANC visits were more likely to deliver under the care of SBAs than those who had attended none or less than four visits. Our findings show that women who had their first or second delivery were more likely to deliver with the care of SBAs than those who have delivered 2 or more. This could possibly be attributed to the fact that women who have a history of normal deliveries may consider themselves competent to have unsupervised deliveries.

Furthermore our study shows that women who reside in rural areas were less likely to be assisted by SBAs than those residing in small towns. This could possibly be attributed to lack of access of health facilities both in terms of distance and financial ability to pay for transport. Our findings that women in Rwampara were less likely to choose assistance by SBAs than those from Kashari could be attributed to the fact that the latter had access to more health centres which provide basic emergency obstetric care. Additionally Rwampara has hilly terrain, which presents transport challenges to women seeking skilled birth assistance during childbirth.

To our surprise, 68% of the women in our study reported that they were assisted by SBAs, twice of what was reported to be the case in the last demographic and health survey (DHS) of 2006 [31]. This result is corroborated with the fact in that our study, 67% of the deliveries did occur in the health facilities where nearly all the SBAs are stationed. The explanation for this could be that there have been an increase in utilization of skilled birth attendance services since the last demographic and health survey was held five years ago. However, direct comparisons between our study and the demographic and health survey need to be done with caution. Furthermore the level of 68% women being assisted by SBAs in the most recent childbirth for Mbarara district is still below the MDG 5 country target of 80%, which was the goal set by the Ugandan government by the year 2010 [28].

Based on findings from this study, the authors defend the need to encourage joint decision-making regarding the location of birth as this clearly promotes assistance by SBAs. Similarly the practice of birth preparedness should be encouraged during the antenatal period through opening up antenatal education to include spouses and eventually other community members. Furthermore, our results supports Ahmed et al. [38] who recommend that there is need to improve women’s economic, education and empowerment status. This implies that in order to achieve Millennium Development Goal number five (MDG 5) there is a need for parallel investments to eliminate poverty (MDG 1), improve general education (MDG 2) and strengthen women’s emancipation (MDG 3) which are interrelated goals. However, women and their spouses should be empowered to take joint decisions on the management of pregnancy and childbirth.

### Study Limitations

The childbirth process is a memorable event for the woman in labour and it is highly unlikely that she will not recall the person who assisted her. We believe that if there was recall bias then it was minimal, especially since it occurred within the last 12 months in relation to this study. Taking this uncertainty into account, we find it unlikely that recall bias distorted our findings to any important degree. Selection bias was minimised by the random method used to select 120 villages included in the study. The sample of women in our study, most likely represent the population of recently delivered women in rural Mbarara district. Confounding was controlled for in the analysis by applying stepwise multivariable logistic regression. Probable confounders were introduced into the regression model stepwise and they did not have significant effect on the association between birth preparedness, decision-maker on location of birth and assistance by SBAs. However, as this study was cross-sectional in design, causal inferences cannot be deduced. Any associations identified therefore need to be studied further.

### Conclusion

Our study indicates that a significant proportion of women in rural areas of Mbarara district deliver under the care of SBAs. Analysis also showed significant association between birth preparedness, women’s decision-making on location of birth and assistance by SBAs. Women who take individual decision on location of birth are more likely not to deliver under the assistance of SBAs. Male involvement in decision-making increases the likelihood of women having attendance by SBAs at birth. Higher education, high household income strengthened the relationship between decision maker and assistance by SBAs at birth.

The antenatal care programmes need to shift from the current policy of only targeting women to promoting increased male and community involvement in safe motherhood programmes. Since relatives and friends are influential in determining where pregnant women deliver from, there is need to use media to reach them with maternal health messages. Formation of community clubs focusing on sexual and reproductive health would be one avenue to reach many people with health messages. In order to achieve the health MDGs 4 and 5 there is need for substantial investment in education (MDG 2), poverty reduction (MDG 1) and gender equality and empowerment (MDG 3).
